# Work-related musculoskeletal symptoms among public municipal elementary school teachers in Cuiabá, Brazil

**DOI:** 10.47626/1679-4435-2023-1131

**Published:** 2024-11-14

**Authors:** Fabiola da Cruz Teles, Mariano Martínez Espinosa, Ediálida Costa Santos, Rita Adriana Gomes de-Souza, Ana Paula Muraro, Ronilson Ferreira Freitas

**Affiliations:** 1 Universidade Federal do Mato Grosso (UFMT), Cuiabá, MT, Brazil; 2 Department of Statistics, UFMT, Cuiabá, MT, Brazil; 3 Nursing School, UFMT, Cuiabá, MT, Brazil; 4 Institute of Collective Health, UFMT, Cuiabá, MT, Brazil; 5 Postgraduate Program in Health Sciences, Universidade Federal do Amazonas, Manaus, AM, Brazil

**Keywords:** occupational health, school teachers, cumulative trauma disorders, saúde do trabalhador, professores escolares, transtornos traumáticos cumulativos

## Abstract

**Introduction:**

The demands and conditions of work can lead to development of a range of health conditions, including repetitive stress injuries and work-related musculoskeletal disorders.

**Objectives:**

To assess the prevalence of musculoskeletal symptoms and the working conditions of primary school teachers working for the public municipal education system in Cuiabá, Mato Grosso, Brazil.

**Methods:**

A cross-sectional study was conducted with teachers working for the public municipal education system in the capital of Mato Grosso state. The sample comprised 326 teachers. Data were collected using the Vocal Production Condition - Teacher instrument and the Nordic Musculoskeletal Questionnaire.

**Results:**

The mean age of the sample was 43.01 years and teachers were predominantly female (87.12%), were married or in a stable relationship (62.70%), had postgraduate qualifications (73.93%), worked at one school only (58.95%), and stated that the pace of their working routine was sometimes stressful (59.01%), that they always took work home with them (57.45%), and that there was sometimes stress at work (54.92%), and reported presence of musculoskeletal symptoms during the last 12 months (76.74%).

**Conclusions:**

The highest prevalence rates of musculoskeletal symptoms were observed among married women, those with postgraduate qualifications, those who worked at one school only, and those who had a stressful working routine. It was therefore concluded that mapping working conditions could provide a foundation for reducing the occurrence of musculoskeletal symptoms and improving the health of this population.

## Abstract (pt)

**Introdução:**

As demandas e as condições de trabalho podem levar ao desenvolvimento de diversas complicações de saúde, entre elas as lesões por movimentos repetitivos e os distúrbios osteomusculares relacionados ao trabalho.

**Objetivos:**

Avaliar a prevalência de sintomas osteomusculares e as condições de trabalho de professores do ensino fundamental da rede pública municipal de Cuiabá, estado do Mato Grosso, Brasil.

**Métodos:**

Estudo transversal, realizado com professores da rede pública municipal da capital de Mato Grosso. A amostra foi composta por 326 professores. Para a coleta de dados, foram utilizados os instrumentos Condição de Produção Vocal do professor e o Nordic Musculoskeletal Questionnaire.

**Resultados:**

Os docentes apresentaram idade média de 43,01 anos, com predominância do sexo feminino (87,12%), casados(as) ou em união (62,70%), com nível de escolaridade em pós-graduação (73,93%), que trabalhavam em uma única escola (58,95%), que afirmaram que às vezes o ritmo de trabalho era estressante (59,01%), que sempre levavam trabalho para casa (57,45%), que disseram que às vezes existia estresse em seu trabalho (54,92%) e que referiram presença de sintomas osteomusculares nos últimos 12 meses (76,74%).

**Conclusões:**

As maiores prevalências de sintomas osteomusculares foram entre mulheres casadas, com pós-graduação, que trabalhavam em uma única escola e que tinham um ritmo de trabalho estressante. Assim, conclui-se que o mapeamento das condições de trabalho pode servir como subsídio para reduzir a ocorrência de sintomas osteomusculares e melhorar a situação de saúde desta população.

## INTRODUCTION

Musculoskeletal disorders are a global health problem. Etiology may be related to physical factors, because of repetitive movements, or linked to working in stressful situations or in static or extreme positions for long periods.^1^ In these cases, repetitive stress injuries (RSI) or work-related musculoskeletal disorders (WRMD) may occur, which are recognized as a severe public health problem, causing functional disabilities and worker suffering.^2^

Work-related musculoskeletal disorders are diseases caused by injuries to the musculoskeletal system and encompass a group of inflammatory and degenerative conditions involving muscles, tendons, nerves, and supportive structures that are associated with movements performed in the workplace. These conditions generally affect the upper limbs, including the hands, wrists, forearms, arms, and shoulders, and there is evidence of direct relationships with the demands of work tasks, physical environments, and the way that work is organized.^2^

Work-related musculoskeletal disorders can also be characterized by a silent process, caused by several different cumulative events and dysfunctions that have a direct impact on a worker’s occupational activities. Such events can result in many social and economic issues when compounded by functional disability, impaired productive capacity, and consequent absence from work.^3^

The social, economic, and political changes that are currently occurring, primarily in the workplace, are both rapid and continuous and are throwing up new challenges. Interpersonal relations, the way that work is organized, and working conditions have been identified as potential causes of harm to workers, whether psychological, social, or physical.^4^

The high prevalence of RSI and WRMD have been explained by transformations affecting work and companies over the years, in which work organization has become an important factor in achieving targets and productivity, in particular amplifying the demand for quality products and services and increasing competitiveness.^5^ As a result of these working conditions, workers’ physical and psychosocial limits are not respected and their ability to perform their jobs is reduced because of this.^6^

Poor working conditions and excessive workloads, in addition to increased demands and skillsets and changes affecting the way work is organized, impose excessive wear on workers, and particularly on teachers, who, in this scenario, are directly affected by these changes.^7^

As such, an international study conducted in Gondar, in the Northwest of Ethiopia, revealed that the prevalence of shoulder and neck pain among primary and secondary school teachers was associated with occupational characteristics. The 12-month self-reported prevalence was 57.3%, indicating that musculoskeletal symptoms are a common occupational health problem in this profession.^8^

A study conducted in the Northeast of Brasil published results for presence of musculoskeletal symptoms in teachers, observing that 87.7% of the teachers reported feeling some type of musculoskeletal symptom, such as pain, discomfort, tingling, or dormancy. Of these teachers, 64.3% stated that their symptoms were related to teaching and worsened when they taught.^9^ Also in Brazil, a study in the Southeast of the state of Bahia found an overall prevalence of 24.3% of musculoskeletal disorders among teachers.^10^

It was found that musculoskeletal symptoms significantly impair the quality of life of teachers working for the public primary education system in the state capital of Mato Grosso (Brazil).^11^ Therefore, in addition to assessing the presence of musculoskeletal symptoms, this study also explores the workplace conditions to which teachers are exposed, focusing on their daily occupational characteristics.

Due to the severity of these diseases, in general, they are detected at advanced stages, which can cause absenteeism or temporary sickness leave and, depending on the severity, can cause permanent occupational disability or early retirement. This imposes significant financial burden on the country’s Social Security system, as shown in data from the National Notifiable Diseases System (SINA -Sistema Nacional de Agravos de Notificação) for 2007 to 2016 presented by Pulgas & Santos,^12^ who observed that there was an increase in RSI/WRMD injury/disease notifications among educational staff, rising from 0.96 to 3.22 per 100,000 professionals from 2007 to 2016.

Thus, the objective of the present study was to assess the prevalence of musculoskeletal symptoms and working conditions among public municipal primary education teachers in Cuiabá, Mato Grosso, Brazil.

## METHODS

A cross-sectional study was conducted in 2017 with regular public municipal primary school teachers in the urban area of Cuiabá, capital of the state of Mato Grosso. Data from the 2017 School Census showed that 75 public schools were providing primary education within this system, with 1,317 teachers working at this level of education.^13^ The study included all teachers who were fully active in the profession, excluding those transferred to other roles or on leave during the data collection period.

The sampling strategy was probabilistic, using simple random sampling, with stratification proportional to the size of the strata. The minimum sample size was estimated at 298, which was increased by 35% was to account for possible losses, making a total of approximately 403 teachers.

After approval of the primary project by the Hospital Universitário Júlio Müller Research Ethics Committee (Ethics Appraisal Submission Certificate: 59503916.7.0000.5541; Ethics committee approval: 1.742.299 and 2.179), a pilot study was conducted at a school that had not been selected in the randomized sample selection and then data collection proper was performed from September to December of 2017, for which all teachers at the selected schools were invited to take part. Teachers who signed the free and informed consent form were given a self-report questionnaire and asked to complete it the next day. Participants who did not return their questionnaires after being contacted on two different days were defined as dropouts.

Teachers’ sociodemographic data and job roles and the way their work is organized were surveyed using the Vocal Production Condition (VPC) questionnaire.^14^ The Nordic Musculoskeletal Questionnaire (NMQ)^15^ was used to assess musculoskeletal symptoms, with the aim of facilitating comparison of the results obtained with those of other studies. This questionnaire is not appropriate for clinical diagnosis, but is used for identification of musculoskeletal symptoms and is an important screening instrument.

The NQM presents a body map illustrating the human body divided into nine anatomic regions. The participant is requested to report musculoskeletal symptoms for the 12 months and the 7 days preceding the interview, consultations with a healthcare professional during the previous 12 months, and any constraints on routine activities during the previous year.^15,16^

The sociodemographic data, job role, and work organization variables from the VPC instrument used in this study were sex, age, marital status, educational level, number of schools at which each teacher works, hours per week with pupils, stressful work pace, repetitive work, time for activities at school, taking work home, intense physical effort, often carrying weight, stress at work, and impact of occupational factors on health. The variables related to musculoskeletal symptoms collected using the NMQ, irrespective of body area affected, were symptoms during the previous 12 months and the last 7 days, any constraints on routine activities, and consultations with a healthcare professional, reported by primary education teachers from Cuiabá.

In this study, statistical analyses were performed using the Statistical Package for the Social Sciences (SPSS) version 17.0. Data were expressed as absolute and relative frequencies and 95% confidence intervals (95%CI) were used for inferential analyses. When there was no intercept between the CIs for the categories of a given variable, it was considered that they were statistically significantly different to a given confidence level - in this case, 95%. Thus, when categories do not overlap, the prevalence of one category would be statistically different to that of the other categories considered for a given variable.

## RESULTS

From a total of 403 study participants, 332 teachers returned the questionnaires, 326 of which (80.9%) were adequately completed and were analyzed. The study results relating to the sociodemographic profile of the sample revealed a mean age of 43.01±9.31 years, with a predominance of teachers who were female (87.12%), married or in a stable relationship (62.70%,) and had postgraduate qualifications (73.93%). Additionally, 82.21% lived and worked in different neighborhoods (Table 1).

**Table 1:** Frequencies of sociodemographic characteristics and job roles of primary education teachers, Cuiabá, state of Mato Grosso, Brazil, 2017

Variable/categories	n	%	95%CI
Sociodemographic			
Sex			
Female	284	87.12	82.99-90.55^*^
Male	42	12.88	9.44-17.01
Marital status			
Has partner (married or in a relationship)	200	62.70	57.13-68.02^*^
No partner (single, divorced, widowed)	119	37.30	31.98-42.87
Educational level			
Postgraduate	241	73.93	68.80-78.61^*^
Graduated higher education	82	25.15	20.53-30.23
Started higher education	2	0.62	0.07-2.20
Others	1	0.31	0.01-1.70
Job role			
Number of schools worked at			
One	191	58.95	53.38-64.36^*^
Two or more	133	41.05	35.64-46.62
Hours with pupils per week			
≤ 20	134	42.14	36.65-47.78
21 to 40	144	45.28	39.72-50.93^*^
> 40	40	12.58	9.14-16.73

95%CI: 95% confidence interval.

n = sample size.

* Statistically significant.

With relation to job role, as shown in Table 1, 58.95% of the teachers reported that they worked at one school only and 45.28% reported that they spent from 21 to 40 hours with pupils. It was observed that the prevalence rates of these categories were statistically different to the others, with a 95%CI.

With relation to the way their work was organized, the majority (59.01%) stated that the pace of work was sometimes stressful, 46.84% said that they sometimes found their work repetitive, 48.43% reported that they sometimes had time to perform their activities at school, 57.45% stated they always took work home with them, 37.62% reported that they rarely exerted intense physical effort, 39.56% reported that they rarely carried weight with frequency, 54.92% said there was sometimes stress at work, and 54.86% stated that occupational factors sometimes impacted their health (Table 2).

**Table 2:** Frequencies of characteristics of the work of primary education teachers, Cuiabá, state of Mato Grosso, Brazil, 2017

Variable/categories	n	%	95%CI
Work organization			
Stressful pace of work			
Never	28	8.70	5.86-12.32^*^
Rarely	30	9.32	6.38-13.03^*^
Sometimes	190	59.01	53.42-64.43^*^
Always	74	22.98	18.50-27.97^*^
Repetitive work			
Never	82	25.95	21.20-31.15
Rarely	69	21.84	17.40-26.80^*^
Sometimes	148	46.84	41.23-52.50^*^
Always	17	5.38	3.17-8.47
Time to perform the activities at school			
Never	6	1.89	0.70-4.06^*^
Rarely	31	9.75	6.72-13.55^*^
Sometimes	154	48.43	42.81-54.07^*^
Always	127	39.94	34.51-45.55
Takes work home			
Never	15	4.66	2.63-7.57
Rarely	21	6.52	4.08-9.80
Sometimes	101	31.37	26.33-36.74^*^
Always	185	57.45	51.85-62.91^*^
Exerts intense physical effort			
Never	84	26.33	21.58-31.53^*^
Rarely	120	37.62	32.28-43.19^*^
Sometimes	94	29.47	24.52-34.80
Always	21	6.58	4.12-9.89^*^
Often carries weight			
Never	76	23.68	19.13-28.71
Rarely	127	39.56	34.18-45.14^*^
Sometimes	91	28.35	23.48-33.62^*^
Always	27	8.41	5.62-12.00^*^
Stress at work			
Never	12	3.81	1.98-6.56
Rarely	23	7.30	4.68-10.76^*^
Sometimes	173	54.92	49.24-60.51^*^
Always	107	33.97	28.75-39.49^*^
Occupational factors impact their health			
Never	34	10.66	7.50-14.58
Rarely	48	15.05	11.31-19.45
Sometimes	175	54.86	49.22-60.41^*^
Always	62	19.44	15.24-24.21^*^

95%CI: 95% confidence interval.

n = sample size.

* Statistically significant.

The results for the anatomic areas affected by musculoskeletal symptoms in the previous 12 months and 7 days, respectively, showed that the highest percentages were for teachers’ lumbar region or upper back (46.75 and 24.83%), followed by shoulders (43.93 and 20.75%), lower back (40.94 and 21.55%), hands/wrists (40.91 and 13.47%), and neck (39.47 and 21.96%). With regard to constraints on activities during the previous 12 months, the most frequently reported area of symptoms responsible, by anatomic region, was the lower back (12.08%), followed by the shoulders (11.63%) and upper back (11.26%). With regard to attending consultations during the same period, the highest prevalence rates were for the lower back (13.99%), upper back (13.33%), and shoulders (12.08%).

Among aspects of musculoskeletal health related to presence or absence of musculoskeletal symptoms during the previous 12 months and 7 days, constraints on activities, and consultations with a health professional during the previous 12 months, irrespective of the area of the body affected, presence of musculoskeletal symptoms during the last 12 months and 7 days was reported by 76.74% and 51.94%, respectively. It was found that 30.55% of the teachers had been constrained from normal activities during the previous 12 months and 34.94% had needed to attend a consultation with a health professional during the previous 12 months. The proportions of these categories were statistically different to the others, with a 95%CI, as shown in Table 3.

**Table 3:** Presence or absence of musculoskeletal symptoms during the previous 12 months and lasts 7 days, constraints on activities, and consultations with health professionals during the previous 12 months, irrespective of the area of the body affected, reported by primary education teachers from Cuiabá, state of Mato Grosso, Brazil, 2017

Variable/categories	n	%	95%CI
Musculoskeletal symptoms			
Last 12 months			
Yes	248	76.74	72.77-82.19^*^
No	71	22.26	17.81-27.23
Last 7 days			
Yes	161	51.94	46.22-57.62
No	149	48.06	42.38-53.78
Consultations during the last 12 months			
Yes	109	34.94	29.65-40.51^*^
No	203	65.06	59.49-70.35
Activities constrained during the last 12 months			
Yes	95	30.55	24.47-36.00^*^
No	216	69.45	64.00-74.53

n = sample size

* 95% confidence interval (95%CI) during the previous 12 months and previous 7 days.

## DISCUSSION

In view of the results of this study, it can be observed that the public municipal primary school teachers stated that their working conditions were sometimes stressful or repetitive or they did not have enough time to perform their activities at the workplace, contributing to them always taking work home with them. Other conditions reported included stress at work and factors that impacted health.

Primary education teachers working for the public system in Cuiabá are a relatively young population, with a mean age of 43.01 (±9.31) years, are predominantly female, and the majority are married or in a stable relationship stable and have postgraduate qualifications. A similar sociodemographic profile to the teachers in this study was also observed among the teachers working for the state education system in Londrina, state of Paraná (Brazil), in a population of 978 teachers (92.2%). The average age of interviewees was 41.5 years (standard deviation [SD] 10) and the majority were female (68.5%) and were living with a partner (60%).^7^

It was observed that the majority of studies reported larger proportions of females, which could be related to the process of woman entering the workplace over the years.^17-19^ In Brazil,^20^ as the teaching profession has become devalued, female labor has been incorporated and the profession has becoming predominantly staffed by females.

With relation to educational level, 73.93% had postgraduate qualifications. This high percentage may be because of a desire to expand possibilities in the job market, targeting better salaries, since postgraduate qualifications can contribute to teachers being better valued and winning promotion, enabling better remuneration.^21^

With relation to job roles, the majority of teachers stated that they worked at one school only and spent 21 to 40 hours with pupils per week. These results are similar to those observed in other studies with this professional category^7,8,22,23^ and may indicate that weekly workload overloads impose excessive physical demands on these professionals. It can therefore be recognized that the normal working day of teachers comprises high demand and extra working hours and that these phenomena are potentially capable of affecting the musculoskeletal system.

With regard to aspects of the way that work is organized, high prevalence rates were observed for teachers who considered that the pace of work was sometimes stressful (59.01%) and that work was sometimes repetitive (46.84%). It should be pointed out that these factors may be important in cases of future complications related to musculoskeletal disorders. Similar results related to these aspects are observed, since activities related to teaching are considered stressful and can have consequences for task performance, which are aggravated if work is repetitive, workloads are elevated, and there is not enough time to perform necessary activities.^23^

In the present study, 48.43% of the teachers reported that they sometimes did not have time to perform their activities at school and the majority (57.45%) reported that they always took work home, which can occur because they sometimes don’t have time to perform activities at school. Different results for the variable time to perform activities have been reported elsewhere,^24^ where the majority (55.5%) of teachers reported insufficient time to perform tasks at school.

In the present study, 37.62% of the teachers stated that they rarely had to exert intense physical effort. Different results were observed in another study, in which 44% of teachers considered that their work demanded considerable physical effort, compared to teachers who did not report exerting physical effort.^24^ In this respect, it is important to point out that excessive physical effort can compromise biomechanical demand, particularly when activities are repetitive, which can be important risk factors for a succession of events that could explain emergence of musculoskeletal disorders, particularly among teachers.^25^

With relation to often carrying weight, 39.56% stated that they rarely did so. Similar results were observed in a study in which 40.4% of teachers stated that mechanical loads had “little effect” with relation to carrying teaching materials.^6^ Moreover, another study observed results contrasting with the current study, since 49.1% of the teachers reported that the weight of the materials they had to take to the classroom caused discomfort.^26^ This situation becomes clear when physical loads in the workplace are analyzed, since when environmental conditions are poor, the teacher has to compensate for a lack of infrastructure through excessive use of the musculature.

Analysis of perceived stress at work and occupational factors that impact health identified that the majority reported occurrence sometimes or always. Agreeing with these results, a study conducted in Ethiopia^27^ found that a significant proportion (60.9%) of interviewees reported stress, whether related to income, health, work, or family.

It should be emphasized that these factors are important to teachers’ working conditions and health, especially with regard to presence of stress, since, although it was described as only occurring sometimes, several different studies have shown that mental issues are of greater importance in studies of occupational diseases. As such, stress is part of this group of issues, since it is one of the symptoms that predisposes to more severe mental complications that can lead to constraints on activities in the future. This was also demonstrated in a study^25^ in which more than half of the teachers (57.1%) had significant symptoms of physical or psychological stress, classifying them as the most common stressors at work.

Corroborating the above, Althomali et al.^23^ reported that musculoskeletal pain is the principal cause of work absenteeism and also of early retirement among teachers, limiting their physical and professional functions. These musculoskeletal disorders constitute one of the most common occupational health risks in the workplace.

When describing the prevalence of musculoskeletal symptoms, it is important to point out that initially these prevalence rates were identified on a body map, showing the areas most affected by presence of symptoms, both during the previous 12 months and/or the 7 days preceding the survey.

Studies indicate high prevalence rates of symptoms related to musculoskeletal disorders and a high prevalence of pain specifically.^22,28^ In the present study, for the last 12 months, the areas most affected were upper back, followed by shoulders and lower back, and then wrist/hands. Contributing to these findings, another study reported that the areas most affected by symptoms were upper back, with 58.7%, followed by lower back, with 53.7%, and neck, with 53.7%.^10,26^ Also in line with our results, another study showed that, in an analysis by body area, high rates of pain were primarily identified in the lumbar area (68.8%), shoulders (63.4%), and wrists (56.7%).^29^

Along the same lines, in the analysis of symptoms during the last 7 days, just 24.83% reported presence in the upper back and 21.96% in the neck, followed by 21.55% with symptoms in the lower back and 20.75% in the shoulders. Supporting these results, another study found that 82% of teachers stated they had felt some type of musculoskeletal symptomology during the last 7 days. By body area, symptoms were most prevalent in the lumbar area (52.9%) and lower limbs (29.4%).^28^

It should be noted that prevalence rates were higher for those who had reported musculoskeletal symptoms during the previous 12 months and/or 7 days, irrespective of anatomic region, although the majority stated they had not been constrained from performing activities and had not had to consult with a healthcare professional during the previous 12 months. It is pertinent to mention that persistent presence of these symptoms can lead to functional disabilities. Even though there had not yet been an increase in absenteeism among these professionals, the presence of symptoms already demonstrates that there is physical wear, with direct implications for their health over the long term.

With regard to medical consultations during the previous 12 months, more than one third (34.94%) of the teachers in the present study consulted a health professional. Corroborating these results, Hermann et al.^26^ found that 51.5% of those investigated reported that they had attended a consultation with a healthcare professional because of problems with musculoskeletal symptoms.

With relation to being constrained from activities during the previous 12 months, 30.55% stated they had been constrained during this period. Confirming this result, Fernandes et al.^29^ found that 47.7% of those investigated reported having been constrained from activities during the previous 12 months because of such symptoms.

It was possible to conclude that mapping the demographic variables and working conditions of these teachers can help to explain the presence of musculoskeletal symptoms. However, further studies should be performed to explore and test these possible associations.

One limitation of this study can be identified as its inclusion only of teachers who were actually involved in teaching activities in the classroom at the time of data collection. As such, professionals who were away from work because of health conditions or other reasons may not have been represented in this population.

Notwithstanding, a strong point of this study is the random sample that was representative of the population of teachers interviewed in Mid-West area of the city of Cuiabá, MT, Brazil.

## CONCLUSIONS

It is concluded that the majority of teachers exhibited musculoskeletal symptoms in the lumbar region and upper back, shoulders, and lower back, had been constrained from activities during the previous 12 months, and had attended consultations during the same period because of the presence of symptoms.

It was found that the presence of musculoskeletal symptoms may be a severe health problem among primary school teachers working for the public municipal education system in Cuiabá, since an elevated prevalence of complaints during the previous 12 months and last 7 days was observed.

Therefore, aspects of the health of these teachers and the emergence of symptoms during the previous year suggest that attention should be directed to preventative care, in order to avoid teachers being constrained from activities or absent from work.

It is important to make professionals aware that presence of any type of musculoskeletal symptom is already a sign of wear to the musculoskeletal system and the path to sickness. Therefore, mapping working conditions could serve as a foundation for efforts to reduce musculoskeletal symptoms and improve the health of this population. Moreover, with regard to issues related to occupational diseases, the role of managers should be emphasized in restoration of activities for prevention and rehabilitation of the physical conditions of professionals, providing better environmental conditions at work and effectively contributing to public policies on occupational health.
